# Nanotherapeutic and Nano–Bio Interface for Regeneration and Healing

**DOI:** 10.3390/biomedicines12122927

**Published:** 2024-12-23

**Authors:** Rajiv Kumar, Chinenye Adaobi Igwegbe, Shri Krishna Khandel

**Affiliations:** 1 Faculty of Science, University of Delhi, Delhi 110007, India; 2 Department of Chemical Engineering, Nnamdi Azikiwe University, Awka 420218, Nigeria; ca.igwegbe@unizik.edu.ng; 3 Department of Applied Bioeconomy, Wroclaw University of Environmental and Life Sciences, 51-630 Wroclaw, Poland; 4 Clinical Diagnosis and Investigation (Rognidan), National Institute of Ayurveda, Jaipur 302002, India; drskhandel@gmail.com

**Keywords:** nanotherapeutic, nano–bio tools, nano–bio interface and regenerative therapies

## Abstract

Wound and injury healing processes are intricate and multifaceted, involving a sequence of events from coagulation to scar tissue formation. Effective wound management is crucial for achieving favorable clinical outcomes. Understanding the cellular and molecular mechanisms underlying wound healing, inflammation, and regeneration is essential for developing innovative therapeutics. This review explored the interplay of cellular and molecular processes contributing to wound healing, focusing on inflammation, innervation, angiogenesis, and the role of cell surface adhesion molecules. Additionally, it delved into the significance of calcium signaling in skeletal muscle regeneration and its implications for regenerative medicine. Furthermore, the therapeutic targeting of cellular senescence for long-term wound healing was discussed. The integration of cutting-edge technologies, such as quantitative imaging and computational modeling, has revolutionized the current approach of wound healing dynamics. The review also highlighted the role of nanotechnology in tissue engineering and regenerative medicine, particularly in the development of nanomaterials and nano–bio tools for promoting wound regeneration. Moreover, emerging nano–bio interfaces facilitate the efficient transport of biomolecules crucial for regeneration. Overall, this review provided insights into the cellular and molecular mechanisms of wound healing and regeneration, emphasizing the significance of interdisciplinary approaches and innovative technologies in advancing regenerative therapies. Through harnessing the potential of nanoparticles, bio-mimetic matrices, and scaffolds, regenerative medicine offers promising avenues for restoring damaged tissues with unparalleled precision and efficacy. This pursuit marks a significant departure from traditional approaches, offering promising avenues for addressing longstanding challenges in cellular and tissue repair, thereby significantly contributing to the advancement of regenerative medicine.

## 1. Introduction

Innovating novel preventive and regenerative therapeutics requires a deeper understanding of interdisciplinary fields, such as biology, medicine, chemistry, computer science, engineering, genetics, and robotics. Researchers are actively devising novel strategies to tackle the challenges associated with developing regenerative therapies aimed at healing and regenerating damaged cells and tissues. Various physiological disorders, including protein conformational diseases (PCDs), disruptions in the mitochondria-to-nucleus signaling pathway, trafficking defects, neuronal dysfunction, endothelial dysfunction, diabetes mellitus, age-related macular degeneration, abnormalities in proteasome activity, as well as a plethora of pathogenic infections and ailments, underscore the complexity of human health [1,2,3].

To achieve innovation in regenerative therapeutics, it is imperative to explore various reactions, mechanisms, and nano–bio interfaces. These nanomaterial-based interfaces show potential in reorganizing the mechanisms of cell mechanotransduction and in promoting intracellular signaling cascades inside the cellular milieu [4,5]. Analyzing reaction intermediates, clarifying how nanomaterials affect oxidation–reduction reactions and enzymatic mechanisms, and comprehending the molecular processes behind chemical transformations, are all part of the study of nanoscale materials [6]. Additionally, by investigating the requirements of the cellular environment and the underlying mechanisms of cellular processes, the application and use of these nano–bio tools in regenerative therapies may be accomplished with ease. By strategizing to achieve better formulations and discovering innovative routes, researchers can harness the potential of nano–bio tools to develop preventive and regenerative therapies tailored for the future [7,8].

Bio/nanomaterials have been used to design and develop preventive and regenerative medicines [9,10,11], which can motivate inherent repair mechanisms owing to their genotype or phenotype and cell environmental settings [12]. These bio-inspired hybrid/composite nanomaterials have unique properties for their use in tissue engineering and regenerative therapies [13]. The development of nano–bio-based preventive and regenerative therapies requires a deep understanding of interdisciplinary fields and schemes to discourse the challenges faced in the field.

This review aims to elucidate the intricate cellular and molecular mechanisms underlying wound healing and regeneration, with a focus on the emerging role of nanotechnology, particularly focusing on nano–bio interfaces in advancing therapeutic interventions. Through synthesizing research from diverse disciplines, including biology, chemistry, and engineering, it seeks to provide insights into the potential of nanoscale tools in speaking to the challenges allied with cellular and tissue repair. This review fills a crucial gap by providing insights into recent advancements in regenerative medicine and the claim of nanotechnology in addressing challenges allied with wound healing and tissue repair. It also offers innovative perspectives on how these interactions can be harnessed to develop advanced therapeutic strategies for promoting tissue regeneration and improving clinical outcomes.

## 2. Nanotherapeutics: Regeneration and Wound Healing

This section introduces innovative strategies employing a variety of nanomaterials for wound healing and tissue regeneration, underscoring their transformative potential in regenerative medicine. Nanotechnology integrates insights from diverse fields, including chemistry, engineering, health sciences, materials science, biology, and physics [14]. The world of medicine has undergone a transformation thanks to nanotechnology, which offers new treatment choices that go beyond cutting-edge materials and include tiny, clever instruments and gadgets built of biomaterials. The development of regeneration and wound healing nanotherapeutics is greatly aided by these nanodrug delivery methods and devices, which include polymeric nanoparticles, lipid nanoparticles, inorganic nanoparticles, nanofibrous structures, liposomes, and nanohydrogel [15]. These nanomaterials possess unique properties and versatile applications that make them promising candidates for applications in tissue engineering and wound healing [16]. A wide-ranging comparison of the diverse natures of nanomaterials commonly used in regenerative therapies is provided in Table 1. Recent investigations have examined the antimicrobial properties and wound healing efficacy of silver, gold, and their alloy nanoparticles synthesized through actinobacterial methods [17,18,19,20,21]. Notably, thermoresponsive gels containing gold nanoparticles have been developed as smart antibacterial and wound healing agents [22]. Various metal nanoparticles, including silver, cerium oxide, silica, gold, zinc oxide, titanium oxide, terbium hydroxide, and copper, among others, have been investigated for their potential in wound healing and regeneration [23]. Aurum, platinum and palladium nanoparticles have also been admired for wound healing and have high antioxidant properties [24,25]. Additionally, nanoparticles, like graphene, oxides, silica nanoparticles, copper nanoparticles, superparamagnetic iron oxide nanoparticles, zinc oxide nanoparticles, terbium hydroxide nanoparticles, titanium dioxide nanoparticles, as well as nonmetallic inorganic nanoparticles, have emerged as promising therapies for tissue regeneration and wound repair [26,27,28]. Ceramic nanoparticles [15], bioactive glass nanoceramics [29,30], bioresorbable nanoceramics [31], bioinert nanoceramics [32], magnetic nanoparticles [33], and polymeric nanoparticles [34] have demonstrated substantial potential in wound healing [31,32]. Bioactive inorganic/organic nanocomposites have also been revealed as regenerative remedies for wound healing [35,36]. Of particular note are nanoparticles (NPs) comprising a hydroxyapatite core capable of binding ions, organic molecules and proteins from the adjacent environment, thereby modulating environmental calcium levels and exhibiting the potential to regulate calcium homeostasis in vivo [37,38]. The pH-sensitive calcium-based nanoparticles were investigated for their ability to augment cutaneous wound repair [37]. These nanoparticles display great stability, tunable structures, and large surface areas, making them ideal for improving wound-healing using in vitro and in vivo prototypes [39,40]. They address the intricacy of wound healing, which is a major obstacle in the repair of injuries and tissue regeneration. These nano–bio instruments are the best choice for long-term medication administration since they have built-in drug delivery mechanisms and therapeutic qualities.

**Figure 1:**
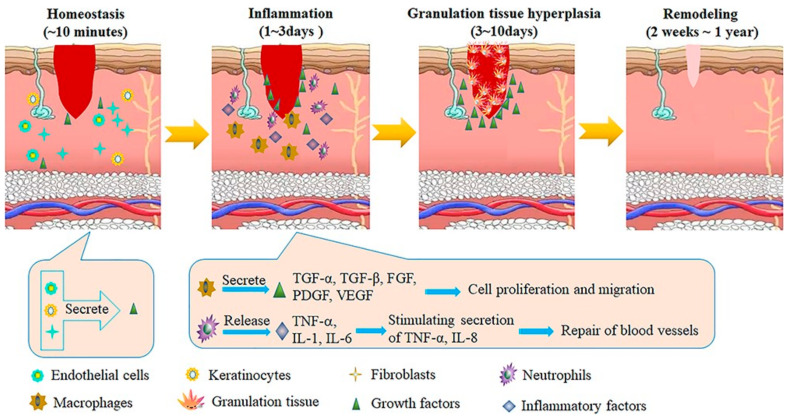
Examples: The processes by which wound injuries heal. VEGF (vascular endothelial growth factor), PDGF (platelet-derived growth factor), TNF-α (tumor necrosis factor alpha) IL-8 (interleukin 8), and FGF (fibroblast growth factor) are examples of growth factors that are transforming growth factor alpha. Reprinted (adapted) with permission from Huang et al. [69].

The practice of wound healing includes the coordinated activation of various cell signaling pathways and types, crucial for its complex physiological nature [67]. Effective antibacterial materials play a vital role in preventing infections at wound sites and mitigating potential complications. Nanotechnology plays a significant role in tailoring therapeutic approaches to the specific requirements of cell mechanotransduction mechanisms. Multifunctional nanomaterials have been explored for treating a range of conditions, including lupus erythematosus, asthma, diabetes mellitus, cancer, Creutzfeldt–Jakob disease, Alzheimer’s disease, Parkinson’s disease, and others [68]. Ongoing efforts focus on innovating therapeutic strategies, particularly in regenerative remedies and vascular tissue engineering, where multifunctional nanomaterials show a promising prospective for diverse biomedical applications. Figure 1 illustrates the wound healing process through its four key stages: homeostasis, inflammation, granulation tissue hyperplasia, and remodeling. These stages involve the secretion and release of various growth factors such as VEGF, PDGF, TNF-α, IL-8, and FGF, which play critical roles in cell proliferation, migration, and the repair of blood vessels [69].

Nanoscale therapeutic devices and tools, such as nanoscaffolds, surface-modified vascular scaffolds and nanoscale devices at the nanoscale, exhibit significant potential for altering cell mechanotransduction processes [70]. They can induce cell alignment, adhesion, and differentiation, thereby facilitating improved endothelial function at the nanoscale [70,71]. Additionally, these instruments find applications in biomedical imaging for the surveillance and monitoring of diseased and injured tissues for preventive interventions [72]. Moreover, they also display nanovibrational characteristics, allowing for the easy distinction between healthy and diseased cells [73]. The on-spot delivery of nanodrugs and biomolecules poses a notable challenge, one that is effectively tackled by these nano–bio tools at the cellular level. Nanodevices/nanocarriers, equipped with nanotherapeutic features, have the ability to impact cell behavior positively, fostering cell regeneration based on phenotype and genotype. Moreover, these nano–bio tools remodel cell mechanotransduction machinery and activate intracellular signaling pathways.

## 3. Nano–Bio Interface, Mechanosensing, Regenerative Therapeutics

Nano/biomaterials have materialized as a new interface in tissue engineering and molecular designing, promoting the proliferation and differentiation of cells [74]. This interface, bridging biomedical and nanomaterial realms, plays a critical role in crafting preventive and regenerative medicine. Such materials catalyze the functionality of tissue-engineered scaffolds at the nanoscale by mirroring the extracellular matrix, thereby augmenting cell adhesion [75]. Additionally, they facilitate the transportation of bioactive components across various cellular compartments, often enhancing immune responses to combat diseases and infections [76]. These tiny gadgets have good medical outcomes and effects, suggesting that their potential should be taken into account while developing preventative and regenerative treatments. The immobilization of functional cell-adhesive ligands may further extend cellular incidents and activate cellular activity for healing wounds and injuries. Surface modification can be used to produce an improved nano–bio interface [77]. Recently, synthetic nanofiber scaffolds that imitate the natural environment have been shown to increase the cellular activity involved in self-healing. The field of regenerative nanotherapeutics is a rapidly developing transdisciplinary field based on the idea that cells have built-in defensive mechanisms [68]. Nanoscale materials and devices are strategically introduced into cellular environments to enhance the performance of cellular organelles, boosting the regular defense mechanisms of the cell and inhibiting the thinning out of diseases and infections [68].

It is possible to see the development of a nano–bio interface as the model for regenerative medicine, which may be used to pave the way for the discovery of nanotherapeutics. By inducing cell signaling and imitating the nano–bio interface, these treatments stimulate the extracellular matrix and facilitate natural communication inside or between live cells. The current requirements and topics to be explored include the surface modification of nanomaterials, tissue engineering scaffold design, nano/biomaterial production, and the nanopatterning of nano–bio surfaces. When building nanoscale tools, these requirements must be upgraded, and intermediary procedures must be altered to reveal and expose innovative production paths for tiny tools. Regenerative bio/nanomaterials and proposed nanoscale instruments may be employed as curative nanomedicines, perhaps through restoring injured neuron cells to promote healing.

**Figure 2:**
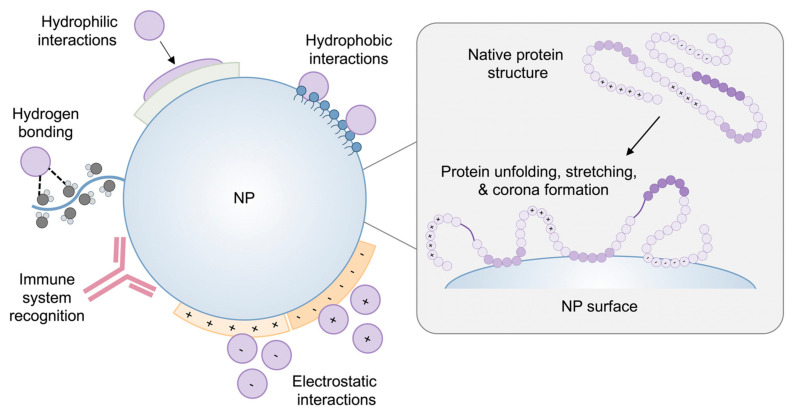
A graphic illustration of the interactions between proteins and NPs, as well as any possible modifications to protein structure on the NP’s surface. Protein function and NP destiny in vivo may be impacted by the NP-induced conformational changes in proteins that reveal cryptic binding sites. Reprinted (adapted) with permission from Bashiri et al. [85].

The principle of motion asserts that reflex actions are natural and involuntary responses to external stimuli, a phenomenon also observed in cellular processes. These responses are mediated by mechanosensors, specialized sensory neurons that detect mechanical forces, facilitating mechanosensing. Mechanosensors serve as the interface between sensory perception and cellular reactions, with mechanical forces playing a pivotal role in initiating cellular responses through mechanotransduction processes [78,79]. Recognizing these cellular processes and alterations in response pathways holds significant promise for advancing theranostics, enabling early diagnosis, disease monitoring, and preventive interventions. This mechanistic framework shares similarities with the concept of symmetry in cellular environments, where mechanical forces induce conformational changes that modulate mechanosensor activity. Typically located within lipid bilayers, mechanosensors possess two distinct ends—one interacting with extracellular matrices or the cytoskeleton, and the other responding to mechanical stimuli [80,81]. These processes generate vibrations within the lipid bilayers, which are then translated into biochemical signals. Mechanical forces induce conformational changes, which subsequently trigger biochemical signaling pathways. These pathways involve various chemical transformations, including protein–protein interactions and enzymatic activities, which are influenced by force-induced structural alterations [82]. It is vital to effectively identify and find chemicals that have since been in the biological environment in order to demonstrate these chemical changes. Their outputs are used in the architecture and design of preventative and regenerative treatments to take advantage of these chemical processes. The interlinked nature of various mechanisms can influence the generation of cellular signaling, with alterations in cell function warranting careful analysis. Within multicellular environments, the responses of cells to adhesion are crucial for the maintenance and repair of adult tissues [83]. The specific characteristics of these microenvironments directly impact a cell’s ability to perceive mechanical stimuli within them [84]. Figure 2 visually depicts the interactions between proteins and nanoparticles (NPs), demonstrating the potential alterations to protein structure on the surface of NPs [85], and highlighting hydrophilic, hydrophobic, electrostatic (represented by the + and − signs), and hydrogen-bonding interactions, as well as immune system recognition.

Comprehending the underlying mechanisms of cell mechanotransduction provides a foundation for devising strategies that enhance the development of preventive and regenerative therapies. This understanding enriches therapeutic approaches and catalyzes the utilization of nanoscale tools in the healing process [86]. Unsolved scientific challenges persist, including the mechanisms by which cells maintain equilibrium amidst the mechanical forces exerted by neighboring cells and how they interpret and convert these forces into biochemical signals. Cadherin, a calcium-dependent protein crucial for cell–cell adhesion, particularly at adherens junctions within multicellular environments, serves as the key component of mechanosensors that initiate sense and a transducer [87,88]. In conclusion, understanding the mechanisms of mechanosensing through mechanosensors is essential for developing effective preventive and regenerative therapies. By addressing unsolved scientific hurdles, researchers can better realize the mechanisms of cell adhesion and the potential applications of these technologies in various fields. Cells use biochemical signals to sense changes in enzymatic reactions or protein–protein interactions, which are influenced by deformations or conformational variations. Cell–cell or cell–extracellular matrix (ECM) proteins act as mechanosensors, sensing mechanical force and conformational alterations and transforming them into biochemical signals. These transformations are often referred to as cellular signaling paths.

The dysregulation of these procedures can lead to the deposition of extracellular matrix proteins, which is believed to be the root cause of aging and other fatal diseases [89]. A dysregulation in cell mechanosensing leads to large-scale complications, damaging cells and tissues, and causing the progression of pathologies. The capability of cells to answer to biochemical signals and reply to variations in the extracellular matrix is crucial for smooth cellular functioning. Understanding the molecular mechanisms involved in crucial adhesion mechanisms and their involvement in cellular sensing within the cell–matrix is essential for achieving success in preventive and regenerative therapeutics. Endothelial cells, for example, can sense pressures in blood flow and transform these mechanical forces into cellular signals [90]. These transmission signals alter the membrane potential and play a vital role in activating kinases, which can trigger oxidative stress, thus initiating the pathways associated with inflammation. Nanoscale tools and devices have the potential to regulate disease initiation and prevent the onset of pathogenesis by establishing checkpoints through nanodevices that control endothelial mechanotransduction signaling pathways. Inflammation is known to instigate pathogenesis [91], and understanding the relationship between the generation of redox signals and endothelial mechanotransduction signaling pathways can provide insights into inflammation processes that impact the entire mechanism [92]. This comprehension is essential for the effective implementation of preventive and regenerative therapies aimed at repairing injuries and wounds in tissues and organs. 

## 4. Signaling Defects, Physical, Chemical, and Biochemical Cues

The goal of controlling cell behavior through nanoscale tools and mimicking the nano–bio interface is a recent theme in research. The creation of new nanoscale instruments that can stimulate the extracellular matrix (ECM) naturally depends heavily on nanofabrication techniques. These nano–bio instruments have the potential to establish useful nanoscale interfaces, which are crucial for regulating cell function. Cells are complex and well-equipped with remarkable features, making it difficult to determine their features. Research is being tremendously aided by the development of nanoscale instruments, which can function at single-molecule resolutions and evaluate data with extreme precision. These methodologies offer the potential for creating nanodevices and nanotools capable of elucidating the intricate cellular environment and emulating the interactions at the cell–matrix interface. Analyzing the composition of cellular compartments and the origins of these interfaces using such methodologies is essential for gaining control over cellular functions. By means of placing a particular emphasis on aspects like cell adhesion, signaling pathways, and biochemical functionalization, researchers can uncover novel characteristics and mechanisms governing cell behavior.

In order to obtain a high temporal and spatial resolution of the cellular microenvironment, fabricated biomimetic scaffolds and nanotools can be utilized to expose cell–matrix and cell–cell interfaces and interactions for vascularization [93,94]. The screening of routes is necessary for successful drug delivery and understanding fluid flow dynamics. Authors plan to design a blueprint for preventive and regenerated therapeutic development, focusing on designing intelligent interfaces to investigate cellular mechanisms and exemplify the essence of regenerative medicine. Analyzing nanotopographical features, cellular pathways, dynamics, intracellular processes, adherens junctions, and mechanosensing are vital components necessary for elucidating cellular functions and therapeutic approaches [93]. Nanotechnology provides sophisticated tools capable of identifying responsive interfaces governing extracellular physical, biochemical and chemical signals through intracellular detection [93]. The emergence of nanoengineered interfaces between cells and nanomaterials, coupled with pathway analysis and the understanding of cellular mechanotransduction, promises significant advancements in precise diagnostics and therapeutic applications, particularly in regenerative medicine.

Nanomachines and nanotools, including nanoparticles, mixed composite scaffolds, and nanotopographies, are effective regenerative therapeutics [95,96]. Utilizing these nanotools and nanodevices for detection enables a comprehensive understanding of the intricate mechanisms governing cellular behaviors and key pathways involved in processes such as adhesion, differentiation, morphology, proliferation, migration, as well as molecular signaling pathways [8,93]. The development of a more effective drug delivery strategy for regenerative medicines for cell regeneration and healing has been greatly aided by these discoveries. Active biological functions are enhanced when nanoparticles and cells interact. For instance, in the human neuroblastoma cell line, gold surfaces with varying degrees of nanoroughness have been used to stimulate neuronal regeneration [97]. The sensing of neurons actively responds to these nanotopography surfaces, enhancing regeneration. The creation of scaffolding materials to mimic the cellular microenvironment and identify physicochemical cues within cells is essential for the repair of damaged organs. Nanostructured surfaces facilitate the early detection of reduced cell adhesion and its consequential effects on cell signaling pathways.

**Figure 3:**
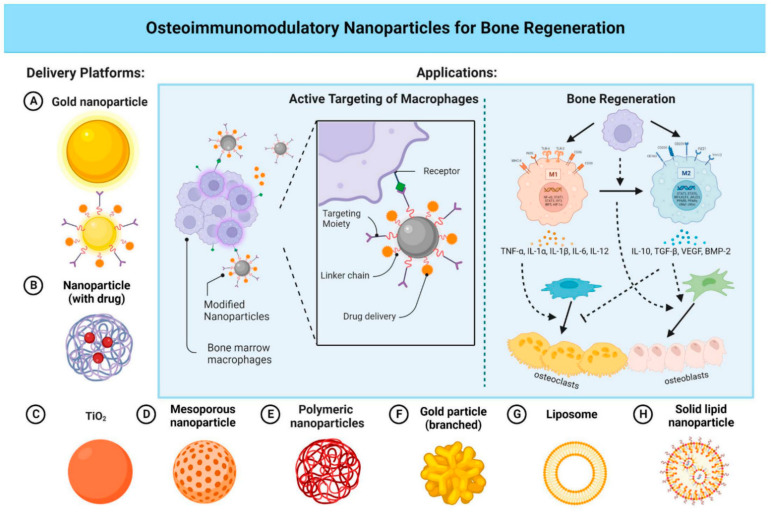
The study suggests that intelligent nanoparticle designs can enhance precision medication effectiveness and expedite clinical translation by overcoming biological barriers and improving distribution strategies. Reprinted (adapted) with permission from Wen et al. [103].

The presence of nanoroughness surfaces disrupts the polarity of neurons during cellular adhesion processes, correlating with increased instances of necrosis and cell death, which are closely associated with the surface roughness of nanostructures [98]. However, the functionality of cell organelles, such as Golgi apparatus fragmentation and nuclear condensation mechanisms, remains unaffected by the presence of nanostructured surfaces. The surface topography of nanodevices and nanotools directly governs cytophilic or cytophobic behavior [93]. Only on flat gold stripes with significant neuronal self-alignment do specific and functional cell attachment occur, suggesting a potential path for the creation of nano/bio-materials that are well-suited to precisely activate biological reactions caused by nanostructures [98,99]. The analysis of mechanotransduction blueprint suggests that cellular mechanosensors can translate mechanical signals into biological signaling [100,101]. Mechanical vibrations can be used to stimulate mechanotransduction, which can be used for drug discovery, regenerative therapeutics, and clinical tissue engineering [102]. Strategies for bone grafting and cell regeneration that are effective include nanovibrational stimulation and the nanoscale sinusoidal vibration approach. These nano–bio kinds of devices are helpful for mechanotransduction studies in other study domains when the backdrop is biologically inert. Wen et al. [103] suggests that intelligent nanoparticle designs can enhance precision medication effectiveness and expedite clinical translation by overcoming biological barriers and improving distribution strategies; these approaches are pivotal for the development of regenerative therapies, which aim to heal injured tissues, restore normal cell functions, and accelerate regeneration (Figure 3).

Regenerative therapeutics aim to heal injured, damaged and wounded cells, tissues, or organs, restoring normal functioning. Nanotechnology fills the gaps in developing nanoscale devices, which enhance the swiftness of the regeneration and recreation of cells and tissues. Nano–bio tools have special topographies that promote the identification of defects in cell and tissue operation and events, such as cellular adhesion, differentiation and migration [8]. Multifunctional nanomaterials and fabrication practices are recommended for developing regenerative solutions. These versatile tools offer promising avenues for treating conditions affecting bone, muscle, cardiovascular, and neural tissues. Nanodevices demonstrate significant potential in initiating and enhancing regenerative processes, aiding in the restoration of normal cellular and tissue functions. Regenerative therapies hold promise as effective interventions for previously untreatable injuries and wounds, offering healing without disrupting existing cellular signaling pathways [104]. Future research endeavors will focus on leveraging nanofabrication, nano–bio technologies, extracorporeal devices, and microfluidic systems to advance regeneration in vital organs, such as the heart, skin, liver and kidneys.

Scaffolds are platforms that are essential for tissue creation because they offer the surface chemicals and connections that encourage cell adhesion and proliferation [105]. Current trends in cell and tissue regeneration include the development of biocompatible and non-toxic nanocomposites. These materials show promise in promoting healing and regeneration within the body. The extracellular matrix, composed of nanoscale components and structures, plays a central role in preventing infections and facilitating the healing process. In response to injuries or pathogenic attacks, the body initiates the healing process promptly to restore proper cellular function and tissue integrity.

Cell–cell and cell–ECM interfaces transpire in cellular compartments, and disrupting these activities can hinder cell fate and functioning. To address this issue, tissue engineering architectures are designed with nanomaterials that can boost cell defense proceedings and regulate cell activities. Nanotechnology has become a pivotal tool in the advancement of defensive and regenerative therapeutics, offering remarkable capabilities in mimicking cellular behavior, addressing signaling deficiencies, and facilitating the regeneration of cells and tissues. The utilization of advanced nanoscale scaffold designs is increasingly advocated for the restoration of damaged cells, tissues, and even organs [106,107,108], playing a noteworthy role in the growth of regeneration therapeutics [109]. The effectiveness of these approaches hinges on their capability and routine as targeted/stimuli-responsive delivery systems, facilitating the precise control over cellular behavior. Nanoscale devices play a crucial role in efficiently modulating cell responses. Regenerative nanodevices address gaps in drug delivery, promoting neurogenesis, axonal growth, and angiogenesis [110]. These nanodevices have potential uses in tissue engineering, medication delivery, cell treatment, diagnostics, and other areas of regenerative medicine [72,111]. Graphene-based materials have shown potential in emerging engineered scaffolds for tissue regeneration, with 3D graphene-based scaffolds showing potential in mimicking cellular events [112,113,114].

The compatibility of these 3D scaffolds relies on the specific structures of the materials utilized in their fabrication, with their efficacy and performance being closely tied to various physical parameters. Through enhancements to the cellular environment at both the physical and chemical levels, these 3D scaffolds play a supportive role in facilitating endothelial matrix function across a spectrum of cellular processes and mechanisms [115,116]. Additionally, they contribute to drug delivery capabilities and exhibit functionalities akin to those found in regenerative medicine applications [117,118]. The 3D scaffold device nanoscale designs and the nature of cell–cell and cell–ECM interactions are compatible and remarkably similar to one another, making integration and coordination easy [93,119,120]. These nanodevices possess crucial features and have become pivotal for the effective execution of regenerative therapy [121]. They exhibit similarities with the extracellular matrix in their composition and configuration, aligning well with the architecture of small-scale tools. Nanodevices are instrumental in transporting nanomedicines for targeted drug delivery, thereby influencing cell behavior and differentiation processes. As a result, nanodevices are key components in tissue engineering and scaffold design and development. Artificial intelligence (AI) holds promise in guiding the selection of optimal scaffold fabrication methods tailored to specific applications [122]. Such advancements facilitate deeper insights into disease pathophysiology and pave the way for the development of enhanced therapeutic strategies.

## 5. Cellular Senescence: Exploring Cellular and Molecular Dynamics in Wound Repair

Wound and injury healing represent intricate processes necessitating advanced therapeutic interventions for both severe and chronic wounds. The journey of healing encompasses several stages, including coagulation, proliferation, and wound remodeling, hemostasis and inflammation, culminating in scar tissue formation. Effective wound controlling stands as a pivotal factor in achieving favorable clinical outcomes. The cellular and molecular underpinnings of wound and injury healing are intertwined with inflammation, innervation, and angiogenesis. The intricate processes of wound and injury healing encompass a network of cellular and molecular mechanisms intertwined with inflammation, innervation, and angiogenesis. Addressing these complexities necessitates the development of innovative therapeutics capable of augmenting regeneration in functional cells and tissues. Central to this endeavor is unraveling the intricate interplay of cell–cell and cell–matrix interactions during injury and treatment, wherein the role of cell surface adhesion molecules emerges as pivotal.

The crux lies in the development of novel therapeutics capable of bolstering regeneration processes within functional cells and tissues [49]. Key features during injuries and treatments revolve around cell–cell and cell–matrix interactions, with cell surface adhesion molecules playing a pivotal role in unraveling the mechanisms governing wound and injury healing, regeneration, and fibrosis [123,124]. Cytokines orchestrate various cellular activities, such as proliferation, matrix synthesis, and migration, alongside other mediators responsible for regulating wound initiation, resolution, and progression [125]. The natural healing cascade initiates promptly post-injury, commencing with hemostasis and the formation of a temporary wound matrix [126,127,128]. The ensuing inflammatory process, known as the coagulation phase, triggers neutrophil recruitment, local fibroblast immigration, monocyte transformation, re-epithelialization at wound edges, neovascularization, and angiogenesis. A comprehensive understanding of these underlying mechanisms is paramount for planning novel therapeutics capable of preempting pathological conditions and fibrosis. Furthermore, considering that elasticity and mechanical heterogeneity can greatly influence wound and injury healing, it is still imperative to shed light on the effects of mechanical forces on the healing epidermis.

**Figure 4:**
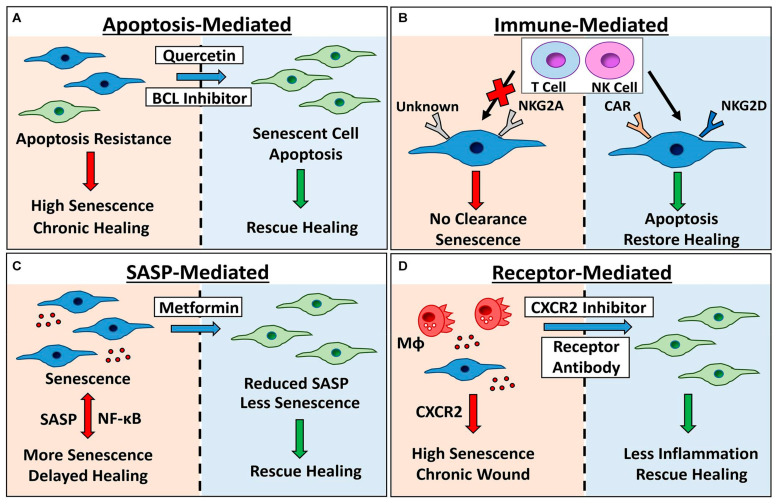
Senescence is therapeutically targeted for long-term wound healing. Senescent cells build up in wounds that heal slowly over time, causing inflammation and inadequate healing. Senescence can be targeted in the following ways: (**A**) to induce apoptosis by blocking pro-survival pathways with BCL inhibitors and broad spectrum drugs (e.g., quercetin); (**B**) to target senescent cell receptors with chimeric antigen receptor (CAR) T cells, or to modulate the expression of natural killer (NK) cell receptors NKG2A and NKG2D to increase clearance; (**C**) to reduce NF-κB-mediated inflammation and bystander senescence by using Metformin or other SASP inhibitors; and (**D**) to inhibit receptors known to potentiate wound senescence (e.g., CXCR2). Bad results are indicated by red arrows and left panels, green arrows/right panels = positive outcomes, MΦ = macrophage, senescent cells = blue cells and therapeutic intervention = blue arrows. Reprinted (adapted) with permission from Wilkinson and Hardman [134].

Skeletal muscle assumes a pivotal role in the regenerative process, driven significantly by muscle plasticity as the initiation point. Integral to this intricate dance of regeneration is calcium signaling, orchestrating various facets of skeletal muscle development, regeneration, and homeostasis. Skeletal muscle assumes a pivotal role in regeneration, with muscle plasticity serving as the initiation point for the regenerative process. Calcium signaling emerges as a critical regulator of skeletal muscle development, regeneration, and homeostasis with calcium dynamics and calcium-dependent mechanisms playing pivotal roles in these processes. Unraveling the dynamics of calcium signaling within the context of muscle regeneration stands as imperative for tailored therapeutic interventions [129,130]. Recent studies have shed light on the role of specific calcium-dependent mechanisms in regulating satellite cell activation and myogenic differentiation, offering potential targets for enhancing muscle regeneration therapies [131,132]. Calcium and calcium-dependent dynamics play a substantial role in these mechanisms. During emergencies, injury-induced calcium signaling navigates through adverse conditions. Epithelial cell components are primarily responsible for deciphering these pathways, which in turn regulate division, differentiation, migration, and cell death. A nuanced understanding of these multifaceted signaling mechanisms is imperative for advancing regenerative medicine. Modern techniques, such as quantitative imaging and computational modeling, hold promise in elucidating the different facets of calcium signaling [133], thereby catalyzing research efforts in regenerative medicine. In the realm of wound repair, calcium signaling predominantly participates in cell migration and regeneration patterns. Hence, the role of calcium and its management assumes significance in wound healing. Notably, the molecular and cellular mechanisms of cellular senescence emerge as critical players in calcium signaling regulation. Cellular senescence stands linked to age-related diseases and serves to steady proliferation seizure induced by various stresses. Calcium signaling emerges as a key driver across all routes of cellular senescence, with alterations in intracellular calcium levels exerting profound effects on cellular senescence. In persistent diseases, cells deploy various strategies within the cellular environment to modulate local conditions, thereby remodeling cellular metabolism. Diminished mitochondrial Ca^2+^ uptake further compounds metabolic remodeling, consequently altering gene expression allied with fibroblast and myofibroblast differentiation routes. Figure 4 illustrates therapeutic strategies targeting senescence for long-term wound healing [134].

Neutrophils, as primary responders to tissue injury, play a critical role in orchestrating the complex route of wound healing [135,136]. Beyond their well-known function in combating pathogens, neutrophils actively contribute to inflammation resolution, debris clearance, and tissue remodeling, thereby facilitating the overall healing process. For instance, neutrophil proteases and the formation of neutrophil extracellular traps (NETs) are critical mechanisms for pathogen elimination and infection prevention [137]. Moreover, the dynamic interplay between neutrophils and keratinocytes, the predominant cell type in the epidermis, is essential for coordinating various stages of wound repair, including hemostasis, inflammation, proliferation, and remodeling [138]. Recent research has elucidated the signaling pathways underlying the pro-inflammatory response during wound healing, offering insights into potential therapeutic targets [139,140,141]. By deciphering these molecular mechanisms, researchers aim to identify novel strategies to modulate the healing process effectively. The intricate crosstalk among chemokines, cytokines growth factors, and other mediators within the extracellular matrix finely regulates cellular behavior and tissue homeostasis. Emerging evidence suggests that extracellular biophysical and biochemical cues play a crucial role in modulating intracellular signaling pathways [142], highlighting the importance of the microenvironment in tissue regeneration and repair.

The integration of cutting-edge technologies, such as quantitative imaging and computational modeling, has revolutionized our understanding of wound healing dynamics [143,144]. For example, recent studies utilizing quantitative imaging techniques have provided unprecedented insights into the spatiotemporal dynamics of cellular responses during wound repair [145,146,147,148], allowing researchers to identify novel targets for intervention. These approaches provide valuable insights into the spatiotemporal regulation of cellular responses and tissue remodeling during wound repair. Still, advancements in bioengineering have facilitated the development of innovative strategies for promoting wound regeneration and enhancing clinical outcomes. A comprehensive understanding of neutrophil function and its interactions within the wound microenvironment is essential for developing targeted interventions in wound healing. By way of leveraging experimental findings and clinical observations, researchers can devise tailored approaches to optimize the healing response and address the challenges associated with chronic wounds.

## 6. Current Trends and Future Perspectives

Regenerative medicine and tissue engineering represent interdisciplinary fields focusing on restoring, maintaining, and enhancing cell, tissue, and organ functions [149]. Nanomaterials and nano–bio tools assume crucial roles in cell and tissue regeneration, supporting cell proliferation and wound healing. The innovation of three-dimensional architectures utilizing nanomaterials and nano–bio tools holds promise in mimicking the extracellular matrix, thereby enabling tissue regeneration and fulfilling other biomedical imperatives. Advancements in nanoparticle design and synthesis stand poised to catalyze the progress in tissue engineering and regenerative medicine [150,151]. Nano–bio tools’ unique features and devices are indispensable for controlling cell and tissue activities at the nanoscale. These tools boast low toxicity, tailored features, targeted/stimuli-response delivery potential, and specific behavior regulation via external stimuli. Regeneration processes mandate high spatial and temporal control, real-time observing, and the delivery of bioactive agents and contrast agents [49,152]. Various nanomaterials, including polymers, metals, ceramics, and their composites, have found application in regeneration. Nano–bio tools resulting from these smart nanomaterials serve as therapeutic modalities to control, regenerate damaged organs, wounds, or cell types, repair, and heal. Multifunctional nanomaterials prove invaluable for formulating small tools and devices to simulate biological paths. Tissue engineering and surface nanopatterning are two applications where nanofabrication may be used to induce certain biological responses in host tissue and cells. The significance of extracellular vesicle-derived cell-to-cell-free treatment for the advancement of regenerative medicine is highlighted. Nanotechnology holds promise in addressing challenges in therapeutic development through its applications in regenerative medicine, wound healing, and tissue repair [111,153,154]. The field of regenerative treatment has advanced thanks to nanotechnology, which has improved tissue and cell regenerating capacities. Subsequent investigations into the remodeling of cell mechanotransduction apparatus will furnish future directives and obstacles in converting findings from the laboratory to the patient’s bedside.

The integration of nanotechnology with biological systems offers unprecedented opportunities for tissue regeneration. By enhancing cell signaling and modulating mechanotransduction pathways, nano–bio interfaces hold enormous potential for promoting tissue repair and regeneration. However, challenges such as biocompatibility and long-term efficacy warrant further investigation. Future research efforts should aim to elucidate the complex interactions between nanomaterials and biological systems, addressing these challenges to unlock the full therapeutic potential of nano–bio interfaces in regenerative medicine. The diverse range of nanomaterials utilized in regenerative therapies present both advantages and drawbacks. While materials like graphene and ceramic nanoparticles show promise in stimulating wound healing and tissue regeneration, concerns regarding their cytotoxicity and immune response modulation necessitate careful consideration. Moving forward, understanding the intricate interplay between nano–bio interfaces and cellular mechanisms is crucial for the development of next-generation therapeutics. Future research endeavors should focus on addressing the limitations of current nanotherapeutic approaches and exploring innovative strategies for enhancing tissue regeneration.

**Figure 5:**
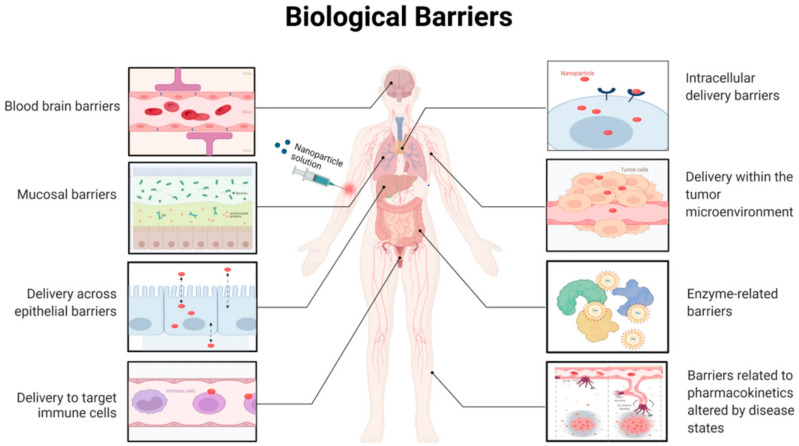
The progressive biological hurdles that nanoparticles must overcome for precise drug administration are covered in this study, with a focus on the significance of designing smarter NPs for increased efficacy and quicker clinical translation. Reprinted (adapted) with permission from (Waheed, Li, Zhang, Chiarini, Armato and Wu [155]).

As illustrated in Figure 5, this study examines the progressive biological hurdles that nanoparticles must overcome for precise drug administration, emphasizing the importance of designing smarter nanoparticles for increased efficacy and quicker clinical translation [155].

## 7. Conclusions

Regenerative medicine stands as an interdisciplinary frontier, leveraging growth factors, scaffolds, biomaterials, and nanomaterials to repair and replace damaged cells, tissues, and organs. This synthesis underscores the intricate interplay between cells and biomaterials within dynamic cellular microenvironments, notably within 3D nanoscale architectures. Through the strategic utilization of nanoparticles, scaffolds, bio-mimetic matrices, and regenerative medicine orchestrate a multifaceted approach to cellular activities, diagnostics, imaging, and targeted treatments, mirroring physiological conditions and enhancing our understanding of in vivo cellular behaviors.

Moreover, the significant strides in regenerative therapeutics underscore the pivotal role of nanotechnology in accelerating tailored interventions. Nanomaterials offer unprecedented precision and efficacy in tissue repair and regeneration, heralding a future where damaged tissues can be restored to their optimal state. While nanotherapeutics have shown remarkable success, it is crucial to recognize their limitations and challenges, including potential toxicity, immunogenicity, and regulatory hurdles. By offering a balanced assessment of both the advantages and drawbacks of nanotherapeutics, this review aims to empower readers with insights to navigate the intricate landscape of regenerative medicine effectively. By elucidating the complex interplay between cellular signaling pathways, extracellular matrix dynamics, and nanotechnology-based interventions, this review contributes to the ongoing dialogue in the field of regenerative medicine.

In addition, it provides a roadmap for future research aimed at overcoming the current challenges in wound healing and tissue regeneration. Researchers in the fields of wound healing, regenerative medicine, and nanotechnology will find this review invaluable for its comprehensive overview of the latest advancements and potential future directions in the pursuit of more effective therapeutic interventions.

## Figures and Tables

**Table 1:** Comparison of nanomaterials used in regenerative therapies.

Nanomaterial	Properties	Applications
Inorganic nanoparticles	High stability, tunable compositions, large surface area [41,42]	Drug delivery, wound healing, tissue regeneration [43]
Liposomes	Encapsulation of drugs, biocompatibility, flexible properties, non-toxic and biodegradability [44,45]	Drug delivery, gene therapy, wound healing [44]
Nanofibrous structures	High surface area, biocompatibility, and high porosity [46]	Tissue engineering, wound dressing, and regeneration [47,48]
Polymeric nanoparticles	Versatile, biocompatible, and tunable properties [49]	Drug delivery and tissue engineering [49]
Lipid nanoparticles	High biocompatibility, great stability, and controlled release [50,51,52]	Drug delivery and gene therapy [53]
Nanohydrogel	High water content, biocompatibility, tunable physical properties, and controllably degradability [54]	Drug delivery, tissue engineering, and wound management [54]
Gold nanoparticles	High biocompatibility, size controllability, and surface plasmon resonance [55]	Imaging, drug delivery, and cancer therapy [56]
Silver nanoparticles	Antimicrobial properties and high surface area [57]	Wound dressing and antimicrobial coatings [57,58]
Graphene	High mechanical strength, electrical conductivity, surface area, and excellent thermal properties [59]	Tissue engineering, biosensors, and cancer therapy [60,61]
Ceramic nanoparticles	Biocompatibility, high surface area/porosity, controlled release, and stability [62]	Bone regeneration, dental applications, and wound healing [32,63]
Bioactive glass nanoceramics	Osteoconductivity and biodegradability [64]	Bone regeneration and wound healing [64]
Magnetic nanoparticles	Magnetic properties, controllable drug release [65]	Hyperthermia therapy, drug delivery, and photothermal therapy [65,66]

## Data Availability

The original contributions presented in the study are included in the article, further inquiries can be directed to the corresponding author.
